# Decomposition and adaptive weight adjustment method with biogeography/complex algorithm for many-objective optimization

**DOI:** 10.1371/journal.pone.0240131

**Published:** 2020-10-09

**Authors:** Wang Chen, Zhao Guohua

**Affiliations:** Department of Mechanical Engineering, Hubei University of Automotive Technology, Shiyan, China; Torrens University Australia, AUSTRALIA

## Abstract

In the EMO (evolutionary multi-objective, EMO) algorithm, MaOPs (many objective optimization problems, MaOPs) are sometimes difficult to keep the balance of convergence and diversity. The decomposition based EMO developed for MaOPs has been proved to be effective, and BBO/Complex (the biogeography based optimization for complex system, BBO/Complex) algorithm is a low complexity algorithm. In this paper, a decomposition and adaptive weight adjustment based BBO/Complex algorithm (DAWA-BBO/Complex) for MaOPs is proposed. First, a new method based on crowding distance is designed to generate a set of weight vectors with good uniformly. Second, an adaptive weight adjustment method is used to solve MaOPs with complex Pareto optimal front. Subsystem space obtains a non-dominated solution by a new selection strategy. The experimental results show that the algorithm is superior to other new algorithms in terms of convergence and diversity in DTLZ benchmark problems. Finally, the algorithm is used to solve the problem of NC (numerical control machine, NC) cutting parameters, and the final optimization result is obtained by AHP (Analytic Hierarchy Process, AHP) method. The results show that the cutting speed is 10.8m/min, back cutting depth is 0.13mm, the cutting time is 504s and the cutting cost is 22.15yuan. The proposed algorithm can effectively solve the practical optimization problem.

## Introduction

In scientific research and production practice, MOPs (multi-objective optimization, MOPs) is of great importance [1]. The conflict among multiple targets in MOPs makes it difficult to get the optimal solution, which is usually a set of compromises. It is called the PS (Pareto optimal solution set, PS) [2]. The corresponding target vector in the target space is PF (Pareto front, PF) [3]. EMO is an important technology to solve MOPs. However with the increase of the number of targets, the existing multi-objective evolutionary algorithms (MOEA) are difficult to solve the MOPs problem for three or more targets. In recent literature reports, MOPs with more than three goals are often referred to as MaOPs [4].

There are many methods to deal with MOPs, but these methods have some limitations. In the process of solving the Pareto optimal solution of practical problems, because the shape of the PF (Pareto front, PF) is very complex, especially when the target has discontinuous PF or low tail peak shape, many subproblems will have the same optimal solution. Recently, soft computing, such as MOPs based on decomposition (MOEA/D) [5], NSGA-III [17], B-NSGA-III, etc., have been applied to solve high dimensional MOPs and have got a better result. Therefore, increasingly scholars have studied the use of soft computing technology to solve multi-objective optimization problems.

With the increase of the number of objectives, the complexity of the MOEA algorithm is increased, and the efficiency of the algorithm is reduced. To tackle the above problems, the decomposition-based methods were design to solve. As one of the popular algorithms, MOPs based on decomposition (MOEA/D) was proposed by Zhang and Li (2007) [6]. MOEA/D is well suited for MOPs because they based on the decomposition strategy for implementing an approximation to the PF. The decomposition strategy and the neighborhood concept were introduced to the methods. The aggregation function is used to compare the solutions and the uniform distribution weight vectors preserving solution convergence and diversity. However, MOEA/D faced the phenomenon called *dominance resistance*. This will lead to a serious deterioration in the selection pressure toward the PF [7]. This is because MOEA/D is used Pareto dominance as selection criterion. The conflicts between diversity and convergence become deteriorated. MOEA/D has low computational complexity, with good convergence and diversity, to get an effective method [8]. However, on the one hand, the weighting vectors used in MOEA/D are generated unevenly. The size N of these weighting vector is not randomly assigned, and N will increase non-linearly as m increases. It satisfies the constraint N = $C_{H+m-1}^{m}$ (where m is the number of targets and H is an integer). On the other hand, the weight vector group should be suitable for solving MaOPs with complex PF, that is, PF is discontinuous or has a shape peak. Qi and Sun [9] et al proposed an improved MOEA/D with adaptive weight vector adjustment to solve the target MOP has a complex Pareto front. Experimental results show that this new method outperforms the benchmark in terms of the IGD, particularly when the PF of the MOP is complex. Dai proposed an improved decomposition-based algorithm to solve mop problems with complex PFs. The proposed algorithm can enhance the diversity of obtained solutions. However, the above literature focuses on the 2-3-objective MOP, and there is little research on MaOPs.

In the previous research [10], the paper designed the BBO/complex algorithm framework based on decomposition to solve MaOPs. The basic assumption of MOEA/D employs a predefined set of uniformly distributed weight vectors. However, when the target MaOPs has a discontinuous PF or has the shape of low tail and sharp peak, several subsystems will have the same optimal solution. In this case, a set of uniformly distributed optimal solutions to PF cannot be obtained by MOEA/D. On the other hand, in the phase of deleting duplicate subsystems, the algorithm could not dynamically adjust the number of subsystems. In this paper, we aim at the refinement of weight vectors and design an adaptive weight adjustment method in MOEA/D to improve BBO/Complex for many objective optimizations. The paper designed the adaptive weight vector adjustment to ensure a certain number of subsystems and increase the diversity of the algorithm to achieve the optimal solution effect. Biogeography based optimization (BBO) is a multi-objective and multi constraint evolutionary algorithm based on biogeography.

BBO/Complex is an evolutionary algorithm based on BBO algorithm, which is oriented to complex system optimization. Generally speaking, the BBO/Complex decomposes a complex system into multi subsystems, and each subsystem contains multi objectives and constraints [11]. When the number of optimization objectives and variables is increasing, the convergence and diversity of the algorithm are poor. The advantage of BBO /complex algorithm is that it is a heuristic algorithm, which is very suitable for complex system optimization. The BBO/Complex algorithm contains many archipelagos, each of which represents a subsystem. Therefore, each subsystem is represented by a group of islands, which corresponds to candidate solutions to the subsystem optimization problem. Selection operator plays an important role in information exchange among the subsystems of BBO/Complex algorithm. Usually, useful information is forwarded to the corresponding subsystem to improve and not mislead other subsystems. Migration phase within the subsystem, the operator selection is usually based on roulette and immigration rate. In the process of cross subsystem migration, the Suitability index variable (SIV) on each emigration island has the opportunity to be replaced by the SIV on the emigration island. However, it is not clear whether the new islands are suitable for these subsystems. Therefore, each subsystem will fall into local convergence and Loss of diversity. In the previous study, the paper designed the BBO/Complex based on decomposition. The algorithm is used to solve the MaOPs. With the further research, the value of weight vector will greatly affect the search efficiency and diversity of the solution when the complex system is divided into several subsystems based on decomposition strategy. A new method based on crowding distance is used to generate a new set of weight vectors, and a new selection strategy is adopted in the process of within migration of subsystems and an adaptive weight adjustment method will change the weight vector value in time, and finally ensure the balance of convergence and diversity of solutions. This paper is based on the above reason and inspired by the decomposition based MOEA/D with adaptive weight adjustment and BBO/Complex algorithm. Our contributions in this work are summarized as follows:

1. The paper have developed a new framework of DAWA-BBO/Complex algorithm for MaOPs, and a new method based on crowding distance is designed to generate a set of weight vectors with good uniformly.
2. The subsystem space obtains a non-dominated solution by a new selection strategy in within-subsystem to enhance the convergence of DAWA-BBO/Complex algorithm.
3. An adaptive weight adjustment method is used to solve MaOPs with complex Pareto optimal front in delete and add overcrowded subsystems by calculating the crowding distance between islands

Section II is related work. The DAWA-BBO/Complex algorithm is described in Section III. In Section IIIV, the paper describe the experiments for the DAWA-BBO/Complex algorithm. In section V, the paper make our conclusions about the algorithm and future research directions are proposed.

## Related work and motivation

### 2.1 Basic definitions

A MaOPs can be stated as:

$$MaximizeF(x)=(f_{1}(x),\ldots ,{\left(f_{m}(x)\right)}^{T}$$ (1)

$$\mathrm{Subject}\;to\;x\in \Omega \subseteq {\mathbb{R}}^{n}$$

Where x = (x_1…_x_n_) ^T^ is the n-dimensional decision variable vector, Ω is the feasible search region, and F: Ω→ℝ^m^ consists of m objective functions, f_i_(x) (i = 1, 2…m), m>3. ℝ^m^ is called the objective space. Accordingly, the set of all Pareto-optimal vectors, EF = {F(x) ∈ℝ^m^ |x∈PS}, is called the efficient front (EF).

### 2.2 BBO/Complex algorithm

**Fig 1:**
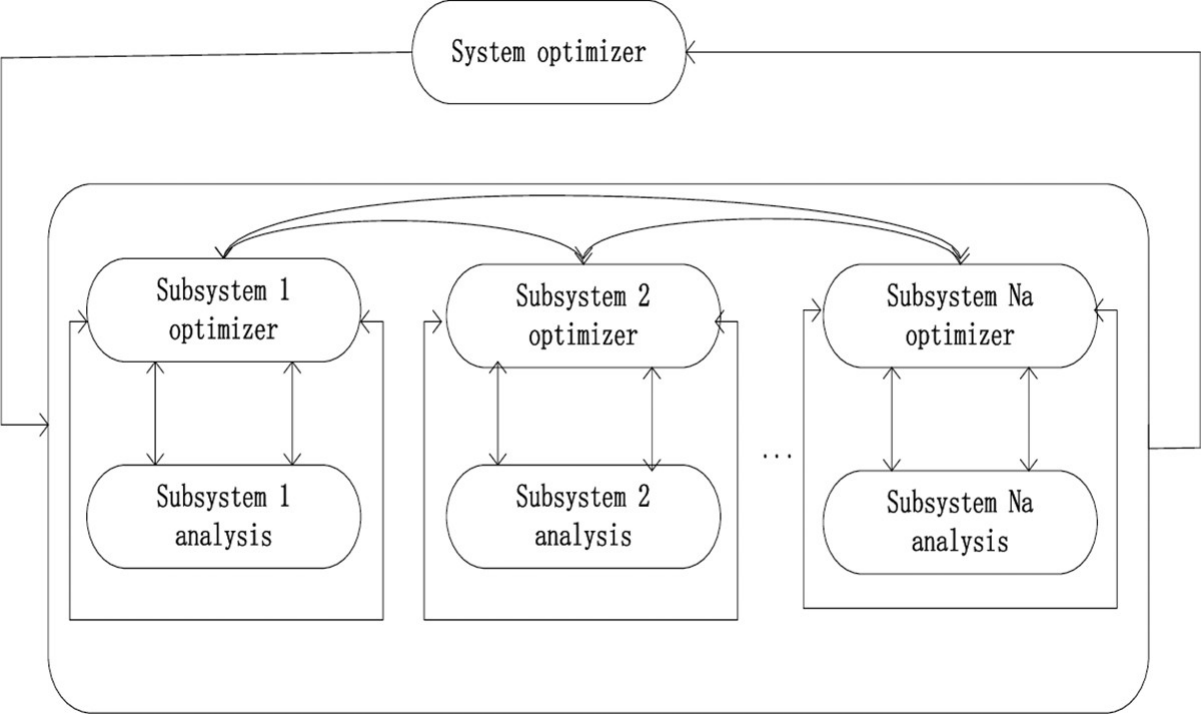
BBO/complex framework.

The complex system can be decomposed into many subsystems by BBO/Complex algorithm, each subsystem contains many objectives and constraints. BBO/Complex algorithm consists of Na islands, where Na represents the number of subsystems in the complex system. There are many islands in the archipelago. These islands represent a candidate solution to the problem. Compared with other MOEA algorithms, the BBO/Complex framework is different from other algorithms. BBO/Complex includes an algorithm framework and optimization algorithm. It mainly provides an effective model for the communication between subsystems and provides a new method for information sharing between subsystems. The BBO/Complex framework is shown in Fig 1.

### Proposed algorithm

#### Framework of the proposed algorithm

DAWA-BBO/Complex uses the original BBO/Complex framework, but develops it for multiple subsystems environments to adapt MaOPs. First, MaOPs are decomposed into multiple subsystems. Firstly, a set of uniformly distributed weight vectors and crowding distance was introduced to decompose the objective space. Then, DAWA-BBO/Complex migration can divide into two categories: within-subsystem and cross-subsystem. In the within-migration, the paper developed a new selection strategy, obtains a non-dominated solution in the cross-subsystem migration, an adaptive weight adjustment method which is mainly consisted by the deleted overcrowded subsystem to make the algorithm to keep diversity. Then the paper perform mutation and clear the duplicate operations.

Algorithm 1 presents the general framework of DAWA-BBO/Complex.

Algorithm 1 The framework of DAWA-BBO/Complex

**Output:** population p

1: Initialization all the parameters

2:    generating parent population, weighting vectors and so on

3:        do decomposition

4:  **while** the termination condition is not met **do**

5:        within-subsystem migration

6:        cross-subsystem migration

7:                mutation and clear the duplicate solution

8:    replace the worst solutions with better islands

9:        **end while**

10:          return p

#### New weight vectors initialization method

The paper set weight vectors such that the optimal solutions of their subsystems are uniformly distributed along the true PF. Most of the approaches for generating weight vectors for different decomposition strategies in MODE/D have been suggested. In this paper, the crowding distance is used on generated weight vectors for the new weight vectors initialization.

For a given set S = {(s_1_, s_2…_ s_m_)| 0<s_i_<1, i = 1…m}, in general, a set of exactly uniformly scattered points in S is very difficult to be found. However, there are some efficient methods than can find a set of well approximately point on S. The Good-Lattice-Point (GLP) [11] method is one of the simple and efficient methods and can generate asset of uniform point to S. A q*m’ an integer matrix G (q, m’) called uniform array is denoted by

$$C(q,m)={\left[G_{ij}\right]}_{q*m}$$ (2)

Where G_ij_-(mod (iu^j-1^, q)) +1, i. q, m’, u is an integer, 2<u<q, mod (iu^j-1^, q) is the remainder of iu^j-1^/ q. Therefore, there are q-1 different integer matrices be generated by these all u. Thus, for given q and m’, they can determine an integer matrix with the smallest discrepancy among their q-1 different integer matrices.

Each row of matrix G (q, m’) determiners a point G_ij_

$$C_{ij}=\frac{2G_{i}{}_{j}-1}{2q},\;i=1∼q,\;j=1-m$$ (3)

Each row of matrix G (q, m) can be defining a point D_i_ = (d_i, 1_, d_i, 1_…d_i, m+1_), i = 1~q. D (q, m+1) can be considered as a set of q uniformly distributed weight vectors.

#### Selection strategy

In within-subsystem phase, if a dominating island is retained in a subsystem, it is likely to be closer to the solution in the adjacent subsystem than two non-dominating solutions in two adjacent sub regions. This solution and its neighborhoods is essential for maintaining species diversity and selecting parents to produce offspring. Therefore, they should be assigned relatively high fitness values. To achieve this goal, the island neighborhood distance is used to calculate the fitness value of the solution in the selection operator. In this way, it is more likely to choose a solution with sparse neighbors to generate a new solution.

The island neighborhood distance can be defined as:

$$IND(isl,Pop)=\Pi_{i}^{k}D_{2}^{nn_{i}^{j}}$$ (4)

Where $D_{2}^{nn_{i}^{j}}$ is the Euclidean distance from the j_th_ Island to its nearest neighbor. I_sl_ is the j_th_ individual island among the population pop.

These new solutions may be the non-dominated solutions to which the sub regional solutions belong. These non-dominated solutions are closer to the real PF. Therefore, this selection scheme is helpful to improve the convergence of the algorithm.

#### Adaptive weight vector

The adaptive weight vector adjustment is mainly consisted by deleting and adds overcrowded subsystem strategy. If the number of deleted subsystem is more than the maximal number of subsystem, the island neighborhood distance of each solution is calculated and the solution with the smallest value of the island neighborhood distance is deleted. Repeat this operation to make the number of deleted subsystems reach the required number. If the subsystem cannot reach the required, the adaptive weight to construct the new subsystem until reaching the required number.

Algorithm 2 Deleting overcrowded subsystem by adaptive weight

**Require:** Check all n SIVs on all k ISI_i_

**Output:** the adjusted population cu_pop

1: **while** there is a duplicated SIV **do**

2:    calculate the island neighborhood distance for each individual ind in the population cu_pop

3:        delete overcrowded subsystem

                **If** the number of deleted subsystem does not reach the required number num **then**

              remove the individual with the minimum the island neighborhood distance

    else return the remaining individual as the cu_pop

The DAWA-BBO/Complex would improve the performance of convergence and diversity through by introducing a new framework for MaOPs. This method will save one significant amount of Central Processing Unit (CPU) time. By these methods, the exploration and exploitation of the DAWA-BBO/Complex algorithm are much improved. The DAWA-BBO/Complex framework is described in Algorithm 3.

Algorithm 3.DAWA-BBO/COMPLEX algorithm

    **1: Initialization with all the parameters. N**_**w**_**, C**_**L**_**, Ls and G**, etc…

        2: **for** g←1 to G do {G is number of generations}

        3: Generating weighting vectors N_W_

        4:Decompose the objective space based on N_W_

    **2: Within-subsystem migration**

        5:Probabilistically select the immigrating islands based on the islands rank,

        6: Probabilistically select the emigrating islands based on the new selection strategy.

    **3: Cross-subsystem migration**

        7:Calculate overcrowded distance between neighbor island from different subsystems

  8:Deleting overcrowded subsystem

  9: update the population

 **4: Mutation**

 **5: Clear duplication**

        10: **if** g>1 **then**

        11: Deleting overcrowded subsystem by adaptive weight adjustment

        11: Replace the worst ISI with the good ISI

        12: update the population

        13: **end if**

        14:**end for**

        15: Display the best populations.

**Fig 2:**
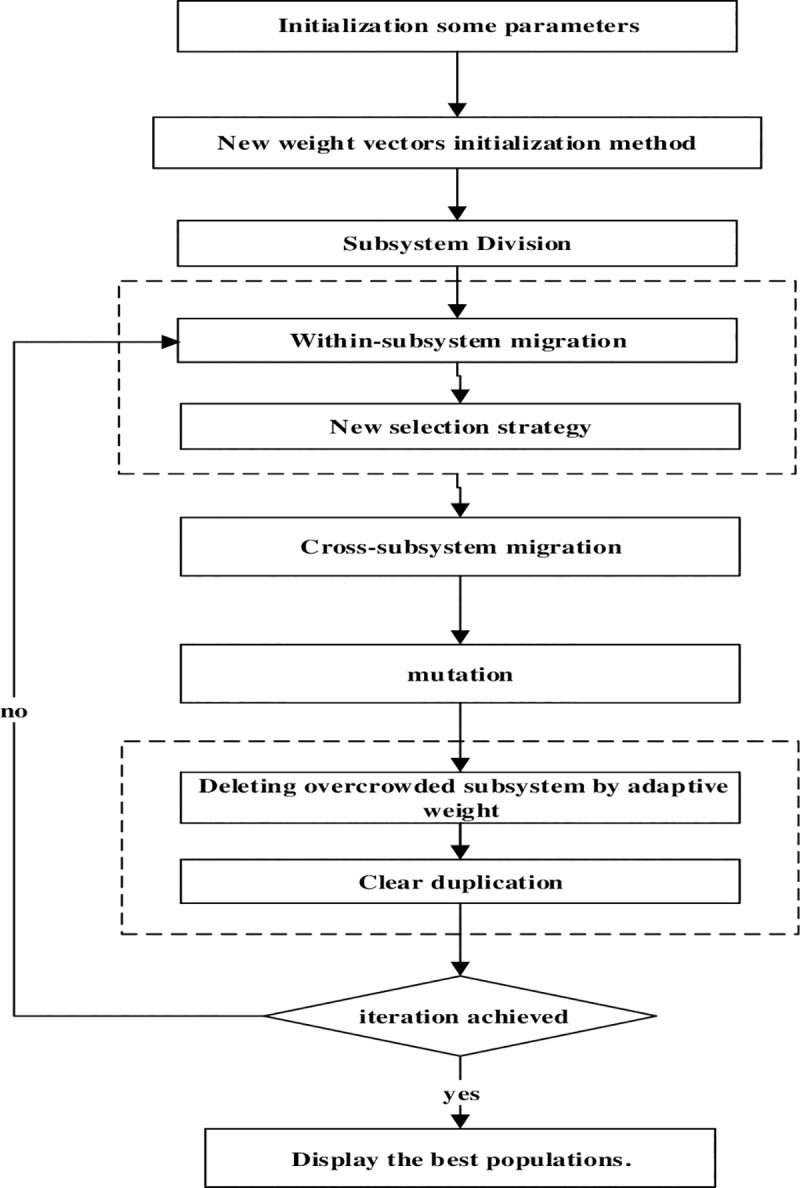
The flow chart of DAWA-BBO/complex.

The flow chart of DAWA-BBO/Complex algorithm is shown in Fig 2.

## Experimental results and discussion

In this section, the DAWA-BBO/Complex algorithm is compared with the best high-dimensional multi-objective optimization algorithm in the near future through experiments. These algorithms, including NSGAIII [12], are based on the Pareto dominance relationship, and the maintenance of population diversity is supported by using a set of evenly distributed reference points. MOEA/D-PBI [13] is the representative of the decomposition based method, which uses a series of predefined weight vectors to keep the diversity of solutions. There is also the BBO/Complex algorithm.

### Test problems

In the EMO test, a number of performance problems have been proposed. For example, the DTLZ test problems [14], and WFG test problems [15] etc. Test functions definitions of DTZ1-DTZ4 functions are listed in Table 1. DTLZ1 is a liner and multimodal function; DTLZ2 is a concave function; DTLZ3 is a concave and multimodal function and DTLZ4 is a concave and biased function. The number of objectives varies are 3, 4 and 5 for each DTLZ problem. The number of decision variables is set as D = m+x-1 for DTLZ test problems, where x = 5 for DTLZ1 and x = 10 for DTLZ2, DTLZ 3 and DTLZ 4. m is the number of optimization objectives of the DTLZ problem.

**Table 1:** Test functions utilized in this experiment.

Name of function	Characteristics
DTLZ1	Liner, multimodal
DTLZ2	Concave
DTLZ3	Concave, multimodal
DTLZ4	Concave, biased

### Quality indicators

In recently, Generational Distance (GD) [16], Inverted Generational Distance (IGD) [17], and Hyper-volume (HV) [18] are classical quality indicators. In the recent study of MaOPs, GD, IGD, and HV can be widely used to test the convergence and diversity of the algorithm, and it has been shown to get good results in testing the MaOPs. The GD reflects the convergence of many objective optimization algorithms. The convergence and diversity of the reflected many objective optimization algorithm by IGD. HV index can measure both the convergence and diversity of the MaOPs algorithm.

a **Generational distance**

Let P* be a set of points evenly distributed over the actual Pareto front, P be the last series of non-dominated points in the objective space. GD reflects the convergence performance of many objective optimization algorithm. The smaller the value of GD is, the better the quality is. GD is described as follows:

$$GD(P,P*)=\frac{\sqrt{\sum_{u\in P}d(u,P*)}}{\left|P\right|}$$ (5)

b **Inverted generational distance**

Let p be the set of solutions obtained by the many-objective algorithm, and let p * be a set of uniform sampling points on the actual effective front (EF). In general speaking, IGD can be used to measure convergence and diversity. The smaller the value of IGD is, the better the solution quality is. Then, the IGD is described as follows:

$$IGD(P,P*)=\frac{\sum_{v\in P^{*}}d(v,P)}{\left|P^{*}\right|}$$ (6)

c **Hypervolume indicator**

HV measures the volume of the dimension area in the target space enclosed by the non-dominated solution set and the reference point obtained by the multi-objective optimization algorithm. The mathematical representation of Super-volume is as follows:

$$HV(p,z)=Volume(U[f_{1},z_{1}]\times \ldots [f_{m},z_{m}],\;f\in A)$$ (7)

P is the final set of non-dominated points in the objective space of many objective algorithms, z = (z_1_, z_2_…z_m_) ^T^ is a reference point in the objective space which is dominated by all Pareto points, and m is the number of optimization objectives.

### Parameters setting

DAWA-BBO/Complex, BBO/Complex、NSGAIII, and MOEAD-PBI in this paper need to set some parameters and they are listed as follows:

1. Population size: The population size N used in this study for DAWA-BBO/Complex is 90, 205, and 208 for three-, four-, and five objective problems, respectively. Furthermore, the size of the NSGA-III population was adjusted as in the original NSGAIII study, i.e., 92, 212, and 276 for three-, four-, and five—objective problems.
2. Parameter set in DAWA-BBO/Complex: set immigration rate is λ_max_ = 1, emigration rate is μ_max_ = 1, m_max_ = 0.1, mutation probability p_bi_ = 0.05, Neighborhood sizes M_HDB_ = 20 and the generation count g = 20. The aggregation function is the Tchebycheff approach [19].
3. Parameter set in BBO/Complex: set immigration rate is λ_max_ = 1, emigration rate is μ_max_ = 1, mutation probability p_bi_ = 0.05, and the generation count g = 20.
4. Reproduction operator setting: crossover probability p_c_ = 1, η_c_ = 30 in NSGAIII. The mutation probability is p_nm =_ 1/V and its distribution index η_m_ = 20.
5. Parameter set in MOEA/D-PBI: Neighborhood size M_MOEA/D_ = 20, probability used to select in neighborhood δ = 0.9. Penalty parameter θ_MOEA/D_ = 5.0.
6. Significance test: The Wilcoxon test is applied with 5% for all pair wise comparisons to test the difference for statistical significance.
7. Stop condition: each algorithm runs 30 independently on each test question. All algorithms are implemented on a 2.6GHz CPU desktop computer, 8GB Random Access memory and Windows 10 operating system. The stop condition of the algorithm is the maximum number of fitness evaluations, as outlined in Table 2.
8. Number of points in Monte Carlo sampling: it is placed at 1,000,000 to ensure accuracy.

**Table 2:** Maximum number of fitness evaluation for different function.

Function	M = 3	M = 4	M = 5
DTLZ1	36500	26400	12600
DTLZ2	22840	42500	73600
DTLZ3	92000	14600	220000
DTLZ4	54700	13200	220000

### Performance comparisons on DTLZ1-DTLZ4 test instance

Tables 3, 4 and 5 show the average and standard deviation of the GD, IGD, and HV values obtained by the four algorithms from DTLZ1 to DTLZ4 with different number of objectives respectively. Addition, it is significant to evaluate the data through common statistical tests.

**Table 3:** Average and standard deviation of GD values obtained by on DTLZ1-4 with four algorithms by different number of objectives.

	M	DAWA-BBO/Complex	BBO/Complex	NSGAIII	MOEAD-PBI
DTLZ1	3	1.1393e-2 (1.02e-1)	6.3223e+0 (1.18e+0) -	1.1290e-2 (2.22e-2) +	1.1157e-2 (1.76e-2) +
	4	5.3423e-2 (2.71e-2)	6.4540e+0 (8.52e-1) -	6.3248e-2 (1.45e-1) -	5.7576e-2 (1.24e-1) -
	5	4.5812e-2 (5.43e-2)	2.1166e+1 (1.74e+0) -	1.4397e-1 (3.23e-1) -	4.9512e-2 (1.33e-1) -
DTLZ2	3	5.2362e-4 (3.26e-5)	6.1273e-4 (2.28e-4) -	5.1238e-4 (1.87e-5)+	7.6754e-4 (3.21e-5) -
	4	2.3145e-3 (5.09e-5)	3.6656e-3 (2.70e-4) -	2.5338e-3 (3.23e-5) -	2.4687e-3 (3.72e-5) -
	5	4.2236e-3 (1.27e-4)	7.3225e-3 (1.01e-3) -	5.1601e-3 (1.40e-4) -	4.5652e-3 (3.10e-4) -
DTLZ3	3	4.1187e+0 (1.33e+0)	6.3071e+1 (4.38e+0) -	2.5970e+0 (1.14e+0) +	4.0375e+0 (1.38e+0) +
	4	3.4722e+0 (2.16e+0)	5.4051e+1 (3.25e+0) -	3.1055e+0 (1.82e+0) +	2.8797e+0 (1.30e+0)-
	5	2.2116e+0 (1.34e+0)	5.8329e+1 (4.35e+0) -	6.8251e+0 (2.59e+0) -	3.8752e+0 (1.39e+0) -
DTLZ4	3	5.1269e-4 (5.41e-5)	1.1234e-3 (4.44e-4) -	5.3425e-4 (1.84e-4) +	3.8285e-4 (4.23e-4) -
	4	2.3517e-3 (1.33e-4)	1.2365e-2 (3.64e-3) -	2.3147e-3 (1.68e-4) +	3.7326e-3 (4.84e-4) -
	5	4.3201e-3 (1.14e-4)	2.3215e-2 (4.31e-3) -	4.4206e-3 (2.45e-4) -	3.5348e-3 (4.89e-4) -
+/-/≈			0/12/0	6/6/0	2/9/1

**Table 4:** Average and standard deviation of IGD values obtained by on DTLZ1-4 with four algorithms by different number of objectives.

	M	DAWA-BBO/Complex	BBO/Complex	NSGAIII	MOEAD/PBI
DTLZ1	3	3.0927e-1 (1.50e-1)	3.0526e+0 (1.44e+0)+	3.0427e-1 (3.24e-1) +	3.0975e-1 (2.42e-1) ≈
	4	2.4459e-1 (2.29e-1)	1.1228e+1 (3.35e+0) -	2.4408e-1 (2.28e-1) ≈	2.2221e-1 (3.01e-1) +
	5	2.5039e-1 (3.24e-1)	2.1438e+1 (2.05e+1) -	2.5021e-1 (3.09e-1) ≈	5.2385e-1 (1.13e-1) -
DTLZ2	3	5.4933e-2 (1.31e-4)	8.4458e-2 (1.71e-3) ≈	5.0928e-2 (4.11e-4) +	5.8549e-2 (4.74e-4) -
	4	1.4070e-1 (2.62e-4)	4.2368e-1 (2.29e-2) -	1.6680e-1 (2.91e-4) -	1.4042e-1 (2.17e-4) -
	5	2.3770e-1 (2.34e-4)	6.3580e-1 (2.85e-2) -	2.3780e-1 (1.01e-4) ≈	2.1329e-1 (2.05e-3) +
DTLZ3	3	1.3606e+1 (1.38e+0)	1.8750e+2 (1.09e+1) -	1.6056e+1 (2.19e+0) -	1.1216e+1 (2.21e+0) ≈
	4	1.2508e+1 (2.50e+0)	2.2400e+2 (2.30e+1) -	1.9181e+1 (2.04e+1) -	1.1817e+1(2.37e+0) +
	5	1.1294e+1 (3.57e+0)	1.3268e+2 (3.14e+1) -	1.4305e+1 (1.03e+1) -	2.7570e+1 (3.13e+0) -
DTLZ4	3	7.1288e-2 (3.93e-2)	1.6542e-1 (1.59e-1) -	5.3035e-1 (3.30e-1) -	7.0259e-2 (2.74e-1) +
	4	1.5173e-1 (1.89e-2)	2.3385e-1 (1.78e-2) -	3.3399e-1 (2.23e-1) -	1.85469e-1 (2.20e-1) -
	5	2.7314e-1 (2.07e-2)	4.0305e-1 (1.43e-2) -	3.3923e-1 (1.38e-1) -	2.8052e-1 (2.51e-2) -
+/-/≈			1/10/1	4/6/2	2/7/3

**Table 5:** Average and standard deviation of the HV values on DTLZ1-DTLZ4 with four algorithms by different number of objectives.

	M	DAWA-BBO/COMPLEX	BBO/Complex	NSGAIII	MOEAD/PBI
DTLZ1	3	3.3969e-2 (3.47e-2)	2.1003e-2 (1.02e-2) -	2.2890e-2 (4.19e-2) +	4.1404e-2 (4.15e-2) +
	4	3.3125e-2 (2.00e-2)	2.1300e-2 (1.31e-2) -	3.3119e-2 (4..1e-2) ≈	4.4275e-2 (2.49e-2) +
	5	2.3760e-2 (296e-2)	2.1213e-2 (2.32e-2) -	3.0544e-2 (5.46e-2) -	2.3785e-2 (4.24e-2) ≈
DTLZ2	3	5.3960e-1 (3.64e-4)	6.8372e-1 (2.39e-2) -	4.3930e-1 (7.58e-4) +	6.3877e-1 (2.29e-3) -
	4	1.6046e+0 (2.67e-3)	2.3026e-1 (1.39e-2) -	2.9872e-1 (7.31e-3) -	2.2026e+0 (2.15e-3) -
	5	1.4284e+0 (3.30e-3)	2.3384e-1 (3.38e-3) -	2.2207e+0 (5.37e-3) -	2.3265e+0 (1.24e-3) -
DTLZ3	3	1.4301e+0 (3.02e+0)	1.4331e+0 (1.21e+0) ≈	1.1295e+0(2.31e+0) +	1.3282e+0(1.03e+0) +
	4	2.6103e+0 (2.12e+0)	2.6004e+0 (1.21e+0) ≈	2.6089e+0 (5.24e+0) ≈	2.6070e+0 (2.75e+0) ≈
	5	1.8089e+0 (3.50e+0)	3.2001e+0 (2.17e+0) -	2.3081e+0 (4.27e+0) -	1.8019e+0 (2.07e+0) ≈
DTLZ4	3	6.9967e-1 (4.67e-2)	7.3880e-1 (3.12e-1) -	7.5007e-1 (5.20e-1) -	8.8247e-1 (1.14e-1) -
	4	8.2690e-1 (3.45e-2)	8.8943e-1 (3.22e-2) -	9.7095e-1 (4.17e-2) -	8.3064e-1 (1.15e-1) -
	5	2.3998e-1 (5.43e-2)	3.5911e-1 (1.26e-2) -	4.1691e+0 (4.14e-2) -	7.8182e-1 (1.24e-1) -
+/-/≈			0/10/2	3/7/2	3/6/3

When the hypothesis required by the paired sample t-test may not be valid, the Wilcoxon test [20] is selected to judge the difference between paired scores at the significant level of 0.05. Wilcoxon test can test whether the proposed algorithm is better ("+"), the same ("≈"), or worse ("-") than other algorithms in each test problem.

The parallel coordinate system is a visual representation of the overall performance of the high-dimensional algorithm [21], in which each row represents a point in the high-dimensional space. If the lines of each object in the graph are distributed in [0, 1], then the better convergence of the algorithm is proved. If the lines can be evenly distributed in [0, 1], then the better distribution of the algorithm is [22, 23].

Table 3 clearly shows that DAWA-BBO/Complex is superior to other algorithms in GD indicators. Only the NSGAIII algorithm is shows better performance in DTLZ4 test problem.

Table 4 shows DAWA-BBO/Complex and NSGAIII show better performance in IGD indicators, and the performance of BBO/ Complex is poor.

As can be seen from Table 5, NSGAIII and MOEA/D-PBI show close performance, overall, the performance of DAWA-BBO/Complex is better than other algorithms.

**Fig 3:**
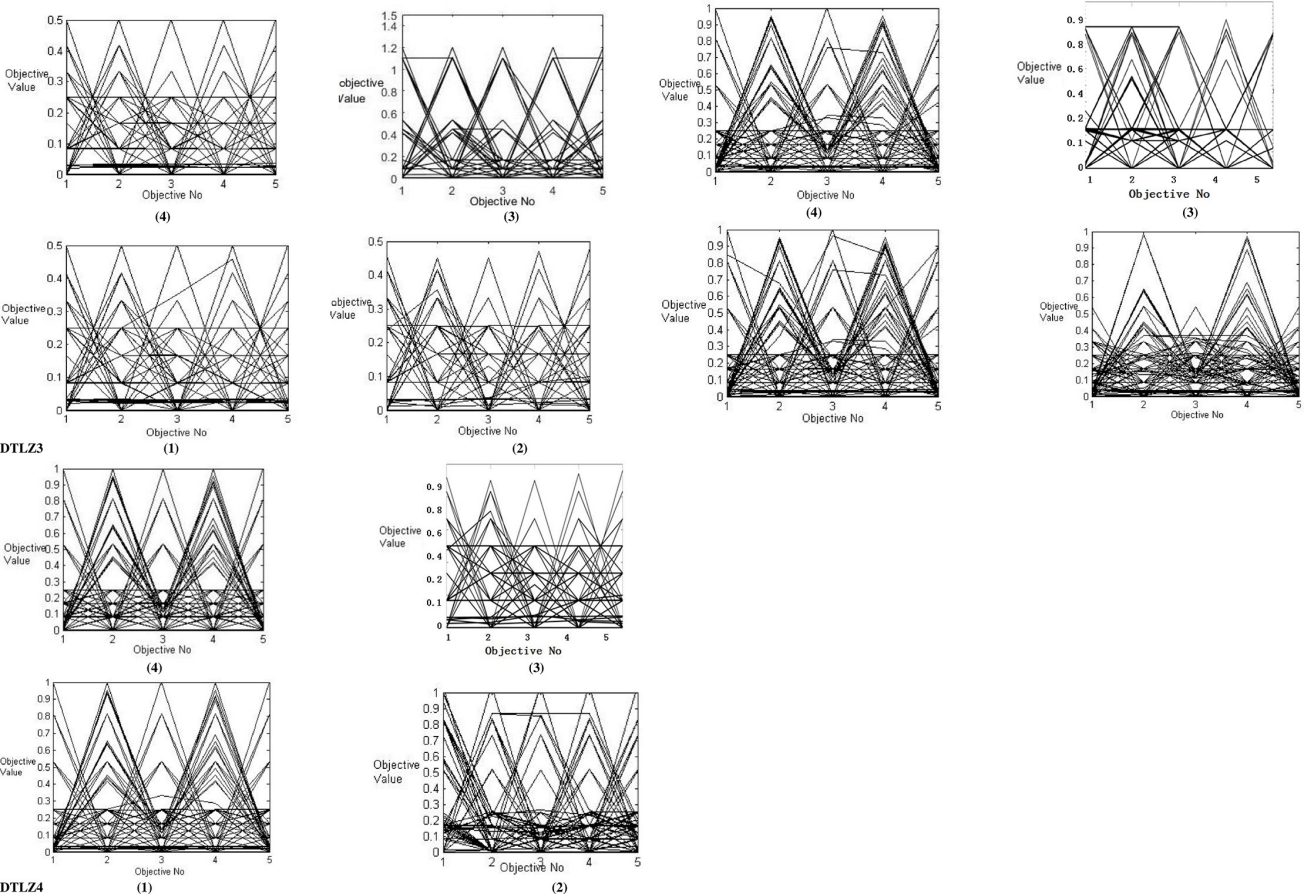
Parallel coordinates figure by four algorithms for the five-objective DTLZ1- DTLZ4. (1) DAWA-BBO/Complex (2) BBO/Complex (3) NSGAIII (4) MOEA/D-PBI.

Fig 3 shows the parallels coordinates of PF obtained by DAWA-BBO/Complex and the other three MOEA with the five-objective of DTLZ1- DTLZ 4. This run is involved in the result closest to the mean IGD value. From this figure, it is found that there is similar performance both in convergence and distribute in DTLZ1 test by DAWA-BBO/Complex, BBO/Complex, NSGAIII, and MOEA/D-PBI.

From the experimental results of DTLZ2, the DAWA-BBO/Complex is better than BBO/Complex and MOEAD/PBI both in convergence and distribute. The convergence performance of NSGAIII is good, but the performance of distribute is poor.

From their plots the results of DTLZ3, the performance of DAWA-BBO/Complex is slightly better than NSGAIII and MOEA/D-PBI; the BBO/Complex is poorly in convergence and distribute. The BBO/Complex is far away from the PF because the maximum value of some objectives is much larger than 1.0.

The performance of convergence and distribute of BBO/Complex is worse than other algorithms. From the experimental results of DTLZ4, it is clear that the DAWA-BBO/Complex is slightly better than NSGAIII, MOEA/D-PBI, and BBO/Complex. When the number of objectives is large, BBO/Complex shows the poor convergence and distribution.

### Parameter sensitivity study

**Fig 4:**
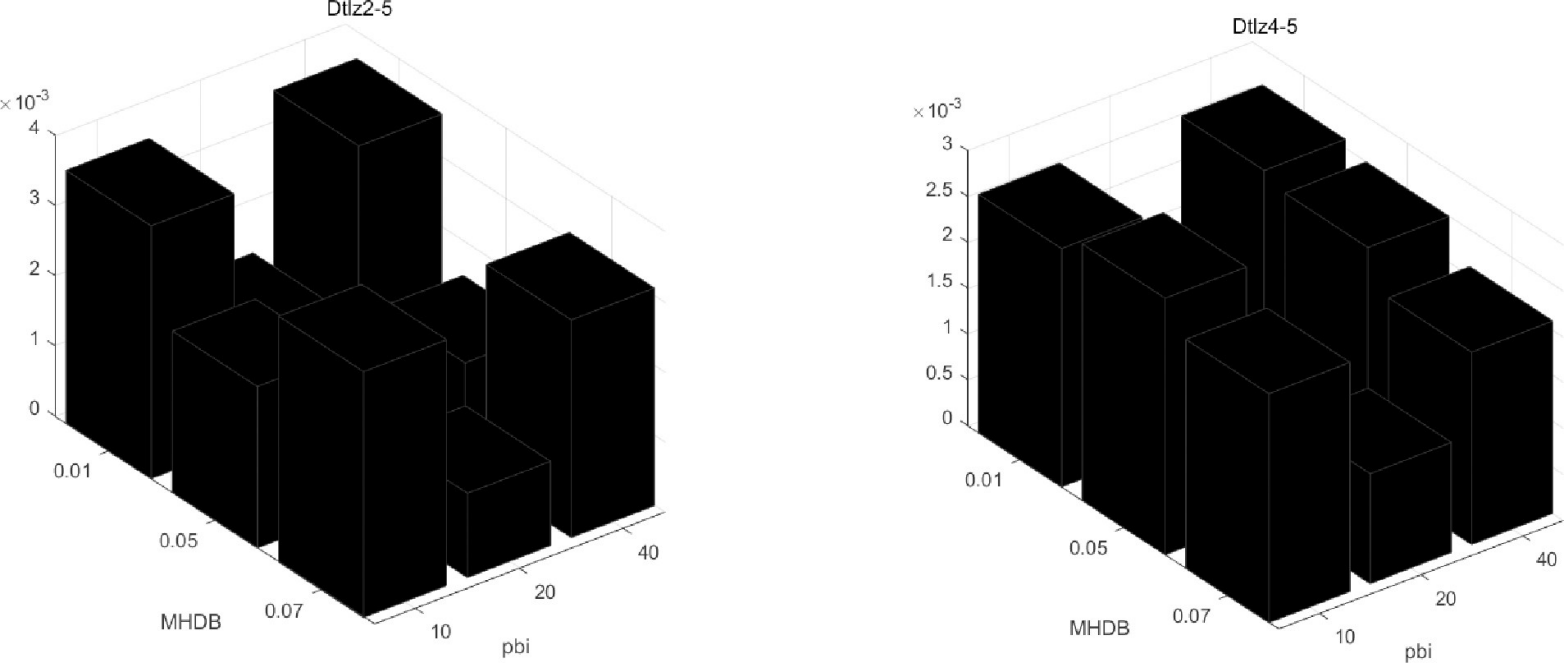
Median IGD values found by DAWA-BBO/Complex with different M_HDB_ and p_bi_ on DTLZ2 and DTLZ4 with five objectives.

For the DAWA-BBO/Complex, M_HDB_ and p_bi_ are very important parameter. In order to study the sensitivity of the algorithm to M_HDB_. The paper discuss 3 values 10, 20 and 40 for M_HDB_ and 0.01, 0.05 and 0.07 for p_bi_. The paper have taken DTLZ2 and DTLZ4 with 5 objectives to compare the performance of all different parameter configurations. In Fig 4, we can see the M_HDB_ = 20 and p_bi_ = 0.05 that is the best choice configuration (The smaller the value, the better the result). When setting M_HDB_ = 10 or 40, p_bi_ = 0.01 or 0.07, the results are worst. The main reason is that repeated operations overuse the local area, resulting in the loss of a lot of useful information.

### Analysis of computational complexity of algorithm

The main purpose of algorithm complexity analysis is to estimate the running time and evaluate the performance of the algorithm from the perspective of computational cost [24]. When the population size is n, the problem objective dimension is m, and the sub-problem domain capacity is M_HDB_, DAWA-BBO/Complex decomposes mop into n sub-problems of scalar optimization. The main computational overhead of DAWA-BBO/Complex is to calculate the aggregation distance o (m (2nlog (2n)) and the sorting of individual non inferior solutions o (m (2n M_HDB_) ^2^). Therefore, the overall time complexity of the algorithm is O (m n M_HDB_). The complexity of BBO/complex algorithm is O (mn^2^), M_HDB_ is much less than n, and so DAWA-BBO/Complex algorithm has better complexity.

### Many-objective optimization of numerical control machine cutting parameters

Numerical control technology is the core technology of advanced manufacturing equipment industry. For manufacturing enterprises, the automatic generation of cutting parameters in the process of NC machining can optimize machining efficiency and quality, improve economic benefits, and enhance the competitiveness of enterprises [25].

The three elements of cutting parameters are cutting speed V, feed F and cutting depth AP. Compared with the cutting speed and feed rate, the cutting depth has little influence on the wear resistance of the tool, and the cutting depth can be determined according to the work piece allowance and specific machining requirements, which can be considered as a known amount. Therefore, the model design variables include cutting speed V and feed rate F.

In the process of NC milling, the maximum production efficiency and the minimum production cost are the optimization objectives. When mass production is carried out, the total working hours for finishing a milling process are T_W_.

$$T_{w}=T_{m}+T_{h}+T_{c}+T_{ot}$$ (8)

T_W_ is the total time to complete a milling process, T_M_ is the actual cutting time of the tool, T_h_ is the average tool change time of a process, T_C_ is the tool change time of each process, T_OT_ is the auxiliary time.

$$T_{m}=\frac{\pi DL}{1000vfZ}$$ (9)

D is the tool diameter; L is the cutting length, v is the cutting speed, f is the feed per tooth of the milling cutter, Z is the number of milling cutter teeth.

$$T_{h}=\frac{\pi LT_{R}}{1000Cv^{\frac{1}{m_{k}}}}v^{\frac{1}{m_{k}}-1}f^{\frac{y_{k}}{m_{k}}-1}a_{e}{}^{\frac{p_{k}}{m_{k}}}z^{\frac{u_{k}}{m_{k}}}a_{p}{}^{\frac{k_{k}}{m_{k}}}D_{}{{}^{1-}}^{\frac{q_{k}}{m_{k}}}$$ (10)

T_R_ is the tool change time, a_e_ is the milling width, a_p_ is the cutting depth, C_V_, k_h_, y_h_, m_h_, u_h_, p_h_, q_h_ are all the tool wear resistance coefficients.

Then the maximum production efficiency function is:

$$\min F_{1}(v,f)=\frac{\pi DL}{1000vfZ}+\frac{\pi LT_{R}}{1000C_{V}\frac{1}{m_{h}}}v^{\frac{1}{m_{h}}-1}f^{\frac{y_{h}}{m_{h}}-1}a_{e}^{\frac{p_{h}}{m_{h}}}Z^{\frac{u_{h}}{m_{h}}}a_{p}^{\frac{k_{h}}{m_{h}}}D^{1‐\frac{q_{h}}{m_{h}}}+T_{C}+T_{OT}$$ (11)

In mass production, the cost of one milling operation is C_p._

$$C_{p}=T_{w}\left(\frac{C_{t}}{T_{0}}+C_{1}+C_{0}\right)$$ (12)

C_t_ is the tool cost; C_1_ is the labor cost per unit time; C_0_ is the management cost per unit time; t_0_ is the tool wear resistance.

Then the minimum cost function is min*F*_2_ (*v*, *f*).

$$\min F_{2}(v,f)=T_{W}(\frac{C_{t}}{T_{0}}+C_{1}+C_{0})$$ (13)

Due to the influence of awesome spindle speed N, feed f, spindle maximum feed force F_fmax_ and work piece quality, the cutting parameters in actual machining should meet the following constraints:

1 Cutting speed constraint:

$$g_{1}(v,f)=\frac{\pi DN_{\min}}{60*1000}-v\leq 0$$ (14)

$$g_{2}(v,f)=v-\frac{\pi DN_{\max}}{60*1000}\leq 0$$ (15)

N_max_ and N_min_ are respectively, the highest and lowest spindle speed of the machine tool; D is the work piece diameter. The cutting speed should meet the spindle speed constraint of the machine tool.

2 Feed restriction:

$$g_{3}(v,f)=\frac{\pi Dv_{f}{}_{\min}}{1000Zv}-f\leq 0$$ (16)

$$g_{4}(v,f)=f-\frac{\pi Dv_{f}{}_{\max}}{1000Zv}\leq 0$$ (17

V_fmin_ and V_fmax_ are the minimum and maximum cutting feed speeds of machine tools, respectively.

3 Cutting force constraint

$$g_{5}(v,f)=\frac{c_{F}a_{p}^{x_{F}}f^{y_{F}}a_{e}^{u_{F}}Z}{N^{w_{F}}D^{q_{F}}}K_{FC}-F_{f\max}\leq 0$$ (18)

F_fmax_ is the maximum force of the spindle and N is the spindle speed. C_F_, X_F_, Y_F_, U_F_, W_F_, Q_F_ and K_FC_ are the Cutting force of parameters. The constraints are turned into an objective. The optimization model for NC machining cutting parameters can be summarized as follows:

$$MinF\;(O_{1},\;O_{2},\;O_{3})$$

$$O_{1}is\;\min F_{1}\;(v,f),\;O_{2}\;is\;minF_{2}\;\left(v,f\right),\;O_{3}\;is\;g_{i}\left(v,f\right)\leq 0,\;i=1,\;2,\ldots 5.$$

All parameters of the algorithm are initialized, and N particles are randomly selected to form islands. Each island is a two-dimensional vector with (V, f) as the variable, corresponding to the feed amount F and the cutting speed V, respectively. The weight vector is generated adaptively. According to the weight vector, minf (O1, O2, O3) is decomposed into different subsystems, then within subsystem migration and cross subsystem migration are carried out, clear duplication by deleting the overcrowded subsystem and add subsystem is carried out, and finally the optimal solution is output. The cutting parameters are shown in Table 6.

**Table 6:** Cutting parameters.

*D*	100	R_amax_	3.23	*T*_R/s_	600	*L*	159
*a*_*p*_	2	*N*_min_	45	*Z*	4	*N*_max_	6000
*r*_*ε*_	1	*V*_fmax_	8000	*F*_*f*max_/N	8000	*V*_fmin_	3
*P*_*cmax*_*/*		(kw·h)	200	*η*	0.8	*a*_*e*_	60

The tool wear resistance coefficient and cutting force coefficient are shown in Table 7.

**Table 7:** Tool wear coefficient and cutting force coefficient.

Tool durability factor		Cutting force coefficient	
*C*_*v*_	1067	*C*_*F*_	7900
*k*_*h*_	0.1	*q*_*F*_	1.3
*m*_*h*_	0.2	*x*_*F*_	1.0
_*qh*_	0.25	*w*_*F*_	0.2
*y*_*h*_	0.2	*y*_*F*_	0.75
*u*_*h*_	0.1	*K*_*FC*_	0.25
*p*_*h*_	0.15	u_F_	1.1

The proposed cost parameter C_T_ = 60 RMB, C_M_ = C_1_ + C_0_ = 2.39 RMB min-1, time parameter T_C_ = 2.39min, tot = 0.1min. In this paper, the Pareto solution obtained by AHP in DAWA-BBO/complex method is used to select the optimal cutting parameter combination. The results are shown in Table 8.

**Table 8:** Final results.

parameter	value
*V*(m/min)	10.8
*f (*mm/z)	0.13
*T(*min*)*	8.24
*C*(rmb)	22.15

## Conclusions

A novel many-objective optimization algorithm called DAWA-BBO/Complex is proposed by this paper. It solved the problem of many-objective optimization which uses the mechanism of decomposition and adaptive weight adjustment based BBO/Complex algorithm. The crowding distance on generated weight vectors for the new weight vectors initialization, the Euclidean distance was used to obtain a non-dominated solution in within-subsystem. The weight adaptive adjustment method proposed in this paper can delete and add overcrowded subsystems by calculating the crowding distance between islands, so as to improve the performance of the algorithm. As it can be seen in the results obtained in experiment, the DAWA-BBO/Complex performance can be enhanced by decomposition-based and control the migration flow of n SIVs between rich and poor islands. The DAWA-BBO/Complex shows a better balance of convergence and diversity for MaOPs. The DAWA-BBO/Complex works well on nearly all the test instances. It is shows the best overall performance on DTLZ problems. DAWA-BBO/Complex can effectively deal with the problem by the number of objectives is 4 and 5, the performance of the algorithm decreased significantly. NSGAIII shows the closest overall performance to the proposed DAWA-BBO/Complex. MOEA/D-PBI gets a good performance on the test problems DTLZ1, DTLZ2, and DTLZ4, but it cannot effectively deal with problem DTLZ 3. BBO/complex perform poorly on all test problems. Finally, a practical case of NC cutting parameter optimization is used to verify the proposed algorithm. Through the modeling of the objective function, constraint processing and algorithm solution, the optimal cutting parameters are obtained by AHP method. The results show that the cutting speed is 10.8m/min, back cutting depth is 0.13mm, the cutting time is 504s and the cutting cost is 22.15yuan. Compared with other algorithms [25], the three cutting elements are further optimized and the cost is reduced.

In addition, in our work, due to the number of objectives less than 6, the paper need to further test the performance of the algorithm when the number of objectives is more than 5. The future work will discuss the DAWA-BBO/Complex in terms of objective number greater than 5 and in solving constrained many-objective problems. At present, the DAWA-BBO/Complex algorithm does not consider the problem of complex system with constraints. The many-objective optimization problem of complex systems with multi constraints is another important challenge faced by MaOPs, which needs further research to solve more problems. In addition, we also hope to use DAWA BBO/Complex to solve more practical problems.

## Supporting information

S1 File (DOCX)

## List of symbols

EMO: Evolutionary multi-objective optimization
MaOPs: Many-objective optimization problems
MOPs: Multi-objective optimization problems
BBO: Biogeography based optimization
BBO/Complex: BBO for complex systems
MOEA: Multi-objective evolutionary algorithm
PS: Pareto optimal solution set
PF: Pareto front
EF: Efficient front
MOEA/D: Multi-objective evolutionary algorithm based on decomposition
SIV: Suitability index variable
ISI: Island suitability index
M: The number of objectives
D: Decision variables
N: initial number of population
N_a_: Number of subsystems
P: Non-dominated of solutions
N_s_: Number of subsystems population
P_d_: Probability distribution
N_w_: The number of weight vectors
L_S_: The number of clusters
E (i): Neighborhood set of weight vectors
G: Number of generations
λ_i_: Immigration rate
μ_i_: Emigration rate
m_i_: Mutation rate
m_max_: Max mutation rate
M_HDB_: Neighborhood sizes
