# Supplementary material for: Decomposition and adaptive weight adjustment method with biogeography/complex algorithm for many-objective optimization
Source: PLoS One. 2020 Oct 9;15(10):e0240131. doi: 10.1371/journal.pone.0240131 (PMC7546505; doi:10.1371/journal.pone.0240131)
Supplement: S1 File — (DOCX) [file pone.0240131.s001.docx]

Median IGD values found by DAWA-BBO/Complex with different M_HDB_ and p_bi_ on DTLZ2 with five objectives

| 0.0036 | 0.0014 | 0.0036 |
| --- | --- | --- |
| 0.0023 | 0.0013 | 0.0015 |
| 0.0035 | 0.0012 | 0.0031 |

Median IGD values found by DAWA-BBO/Complex with different M_HDB_ and p_bi_ on DTLZ5 with five objectives

| 0.003 | 0.0035 | 0.0035 |
| --- | --- | --- |
| 0.0028 | 0.0027 | 0.0027 |
| 0.003 | 0.0032 | 0.0035 |
